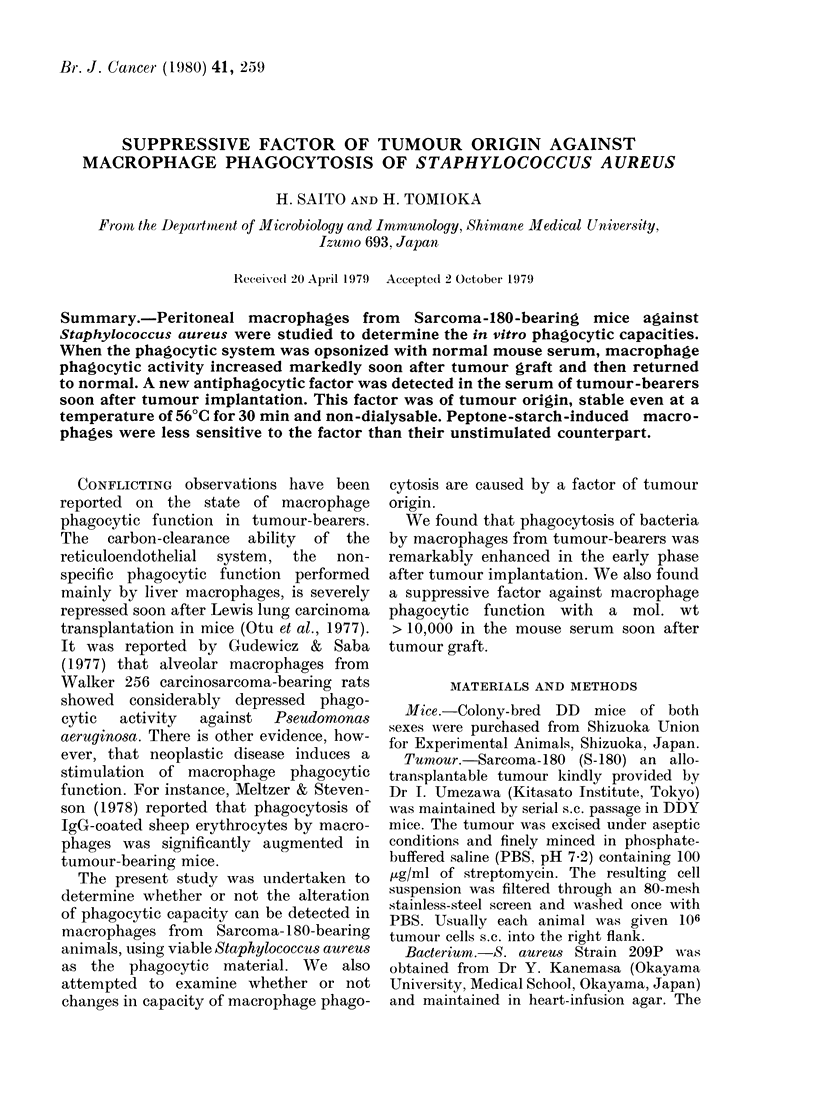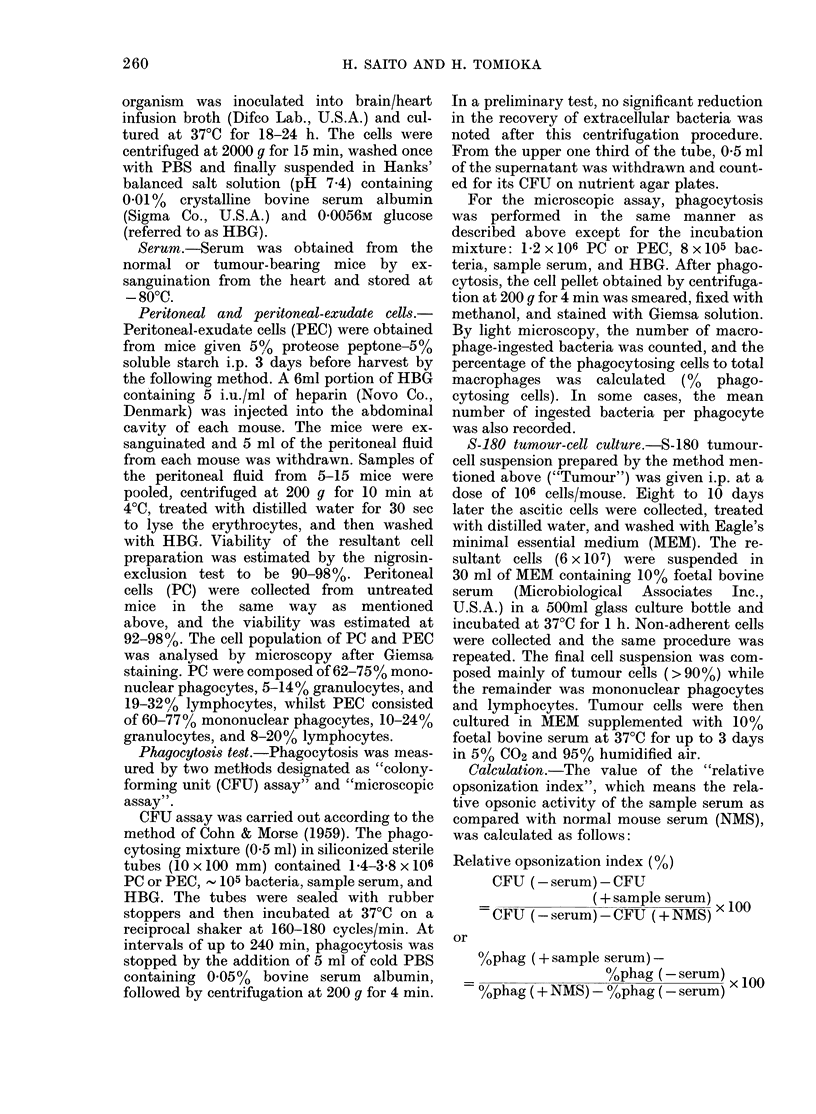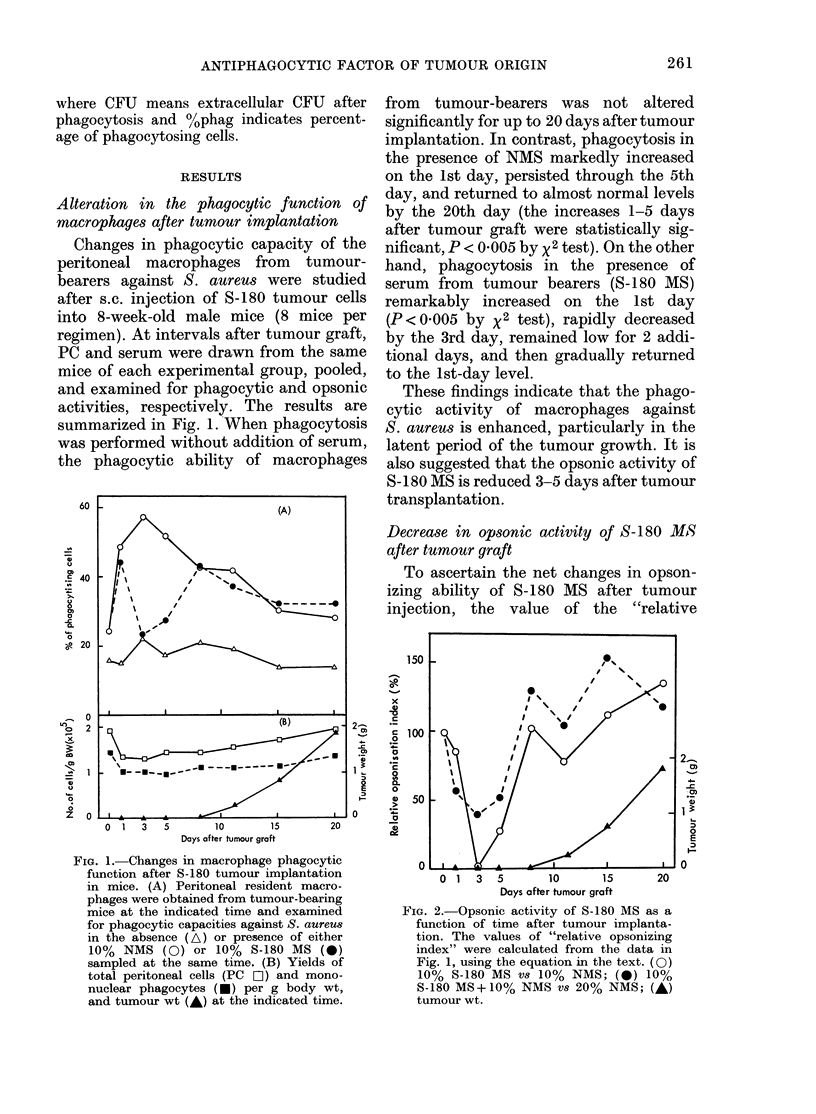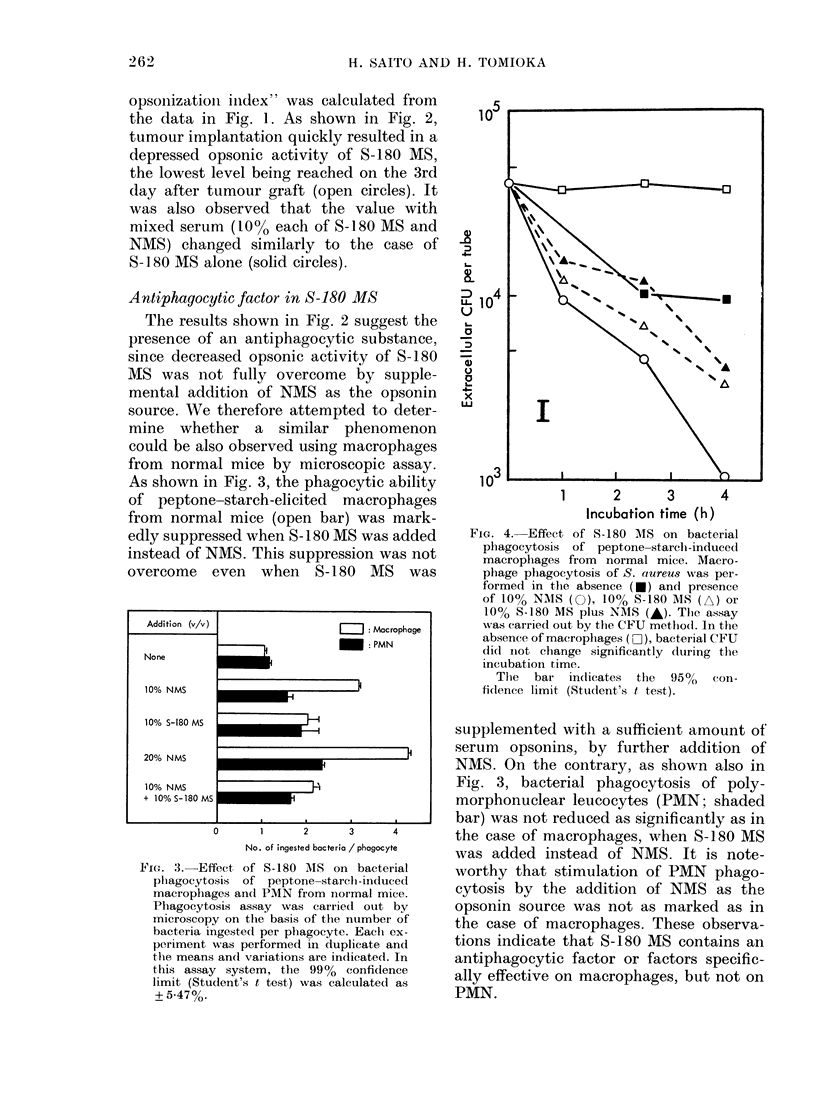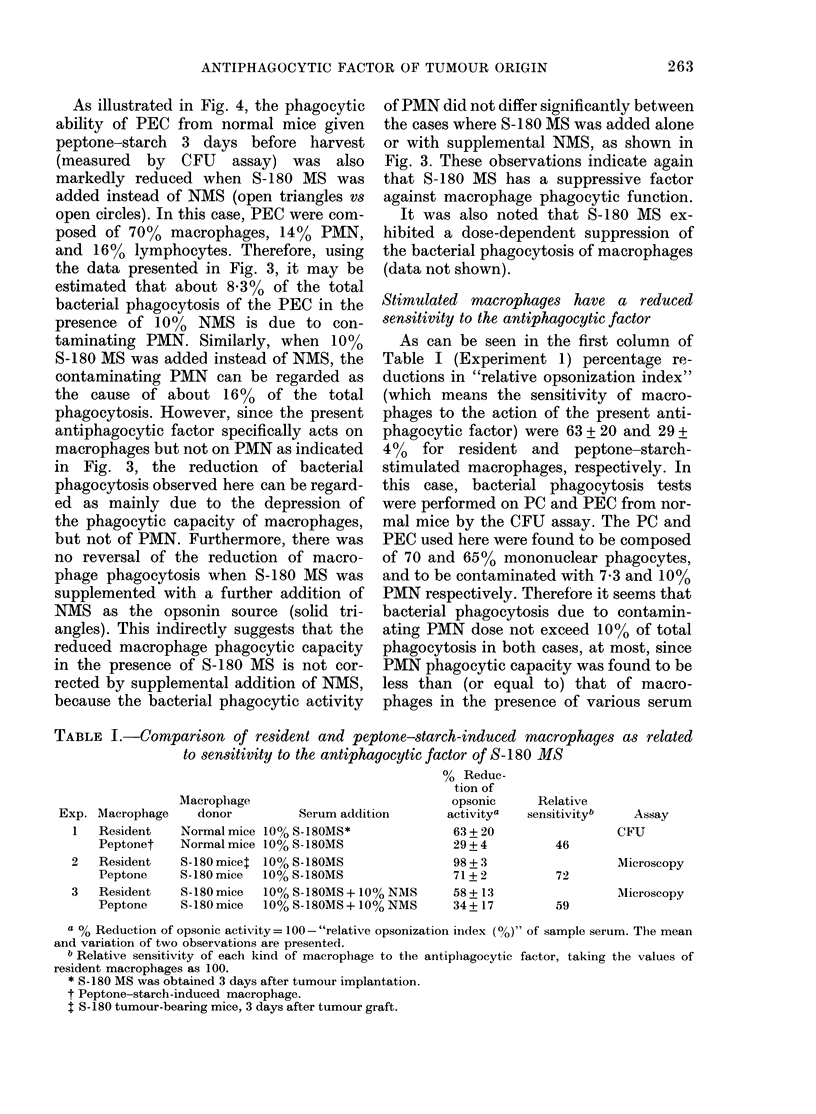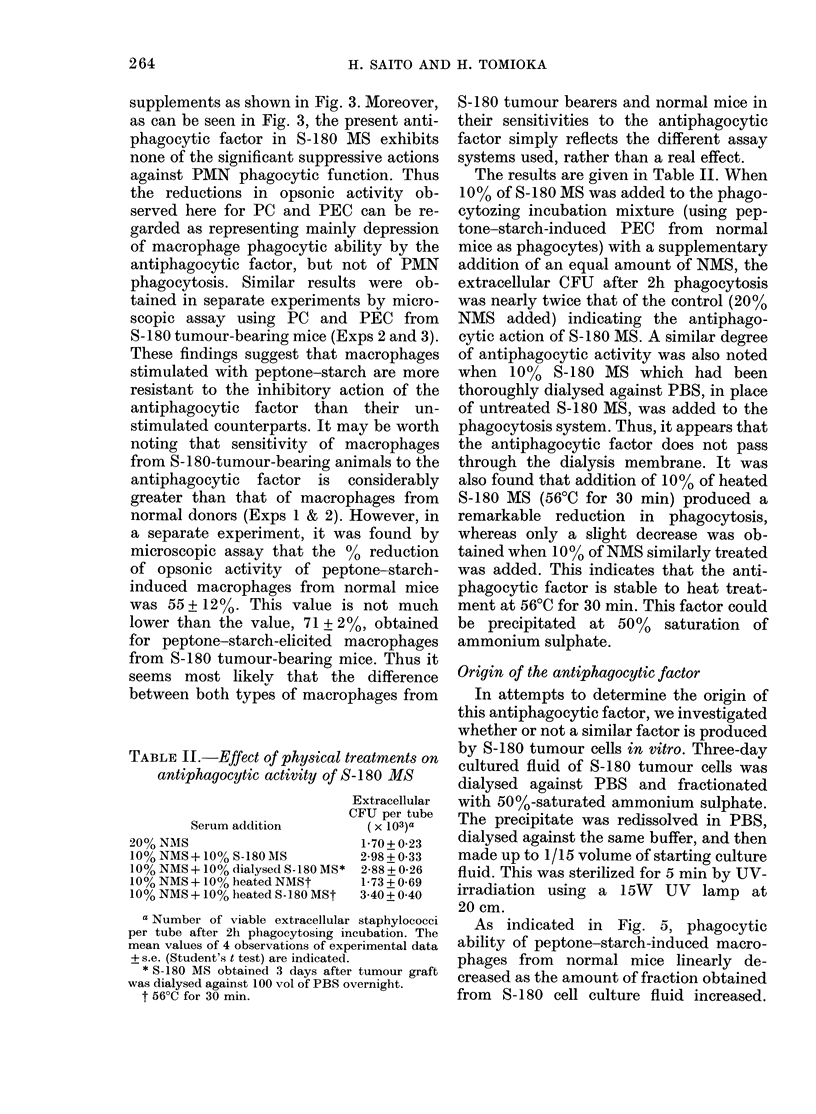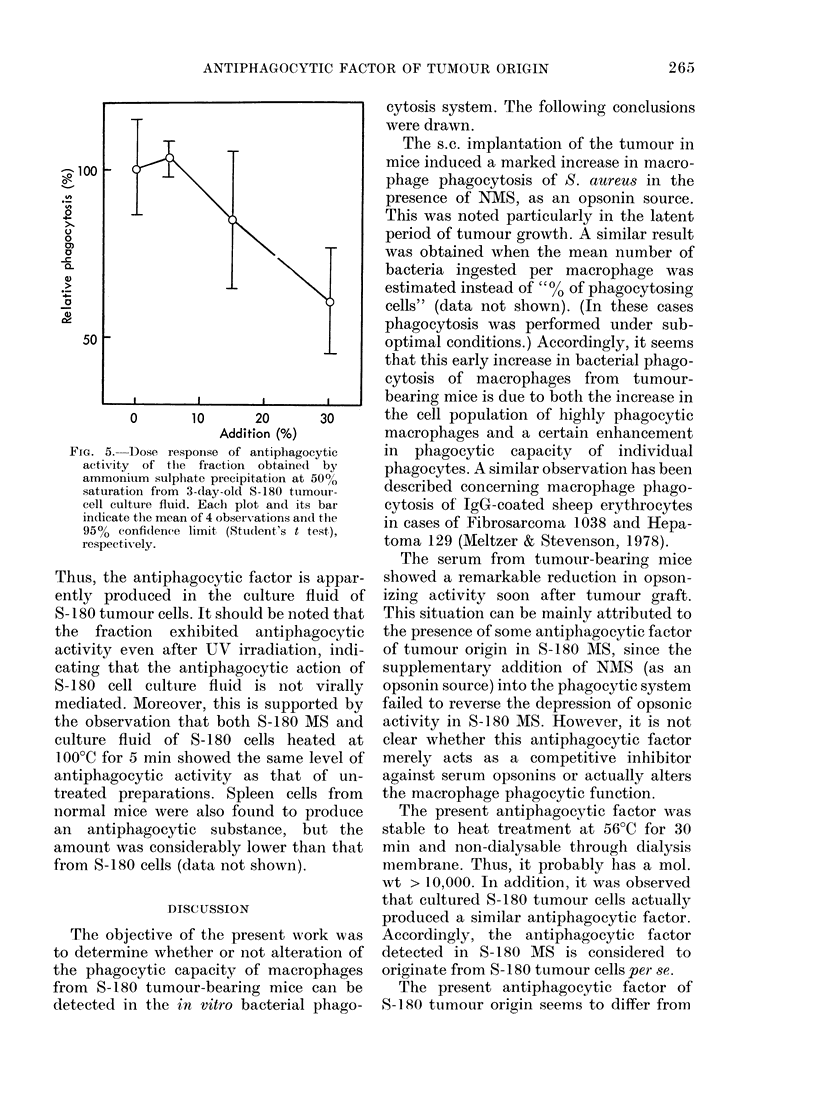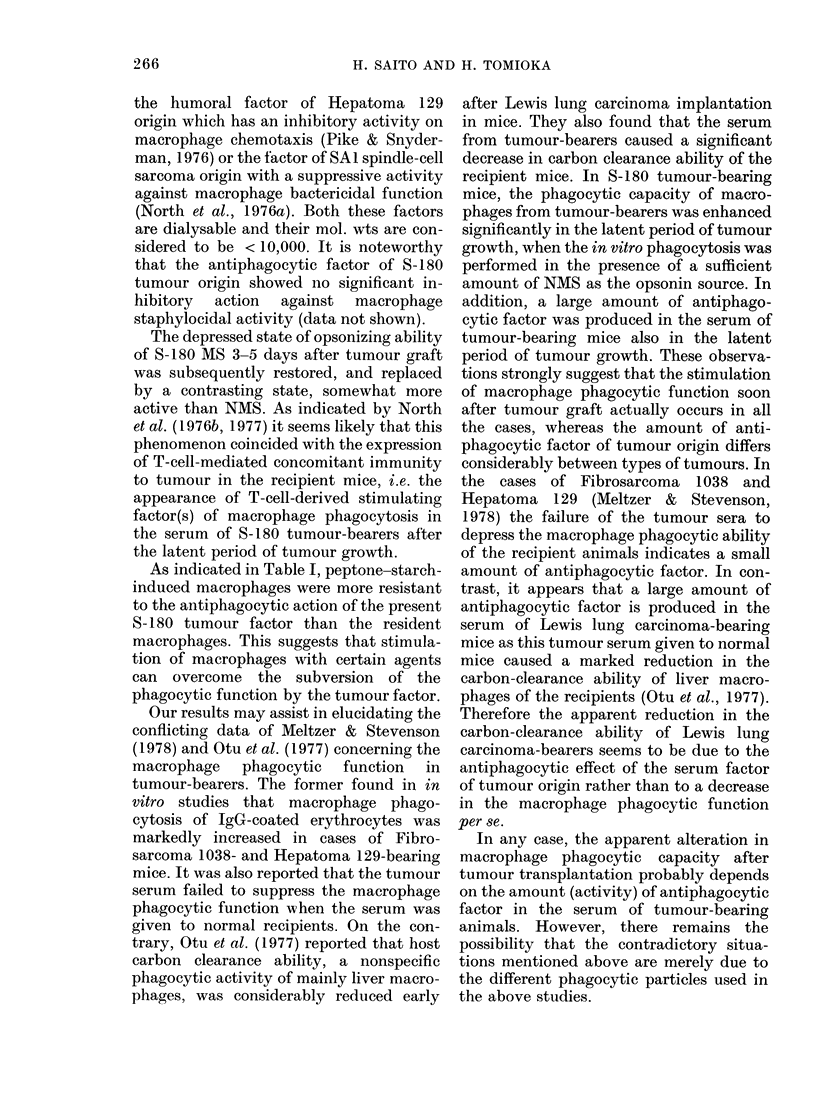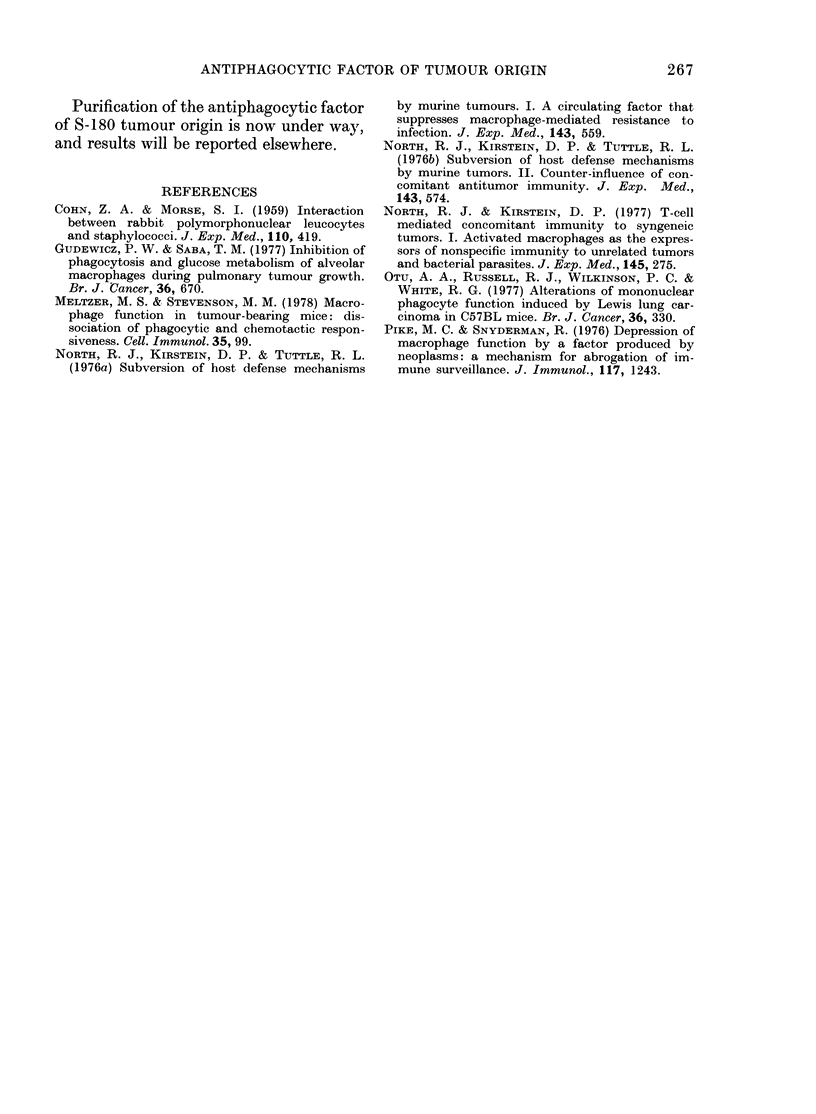# Suppressive factor of tumour origin against macrophage phagocytosis of Staphylococcus aureus.

**DOI:** 10.1038/bjc.1980.38

**Published:** 1980-02

**Authors:** H. Saito, H. Tomioka

## Abstract

Peritoneal macrophages from Sarcoma-180-bearing mice against Staphylococcus aureus were studied to determine the in vitro phagocytic capacities. When the phagocytic system was opsonized with normal mouse serum, macrophage phagocytic activity increased markedly soon after tumour graft and then returned to normal. A new antiphagocytic factor was detected in the serum of tumour-bearers soon after tumour implantation. This factor was of tumour origin, stable even at a temperature of 56 degrees C for 30 min and non-dialysable. Peptone-starch-induced macrophages were less sensitive to the factor than their unstimulated counterpart.


*Br. J. Cancer* (1980) **41**, 259

### SUPPRESSIVE FACTOR OF TUMOUR ORIGIN AGAINST MACROPHAGE PHAGOCYTOSIS OF *STAPHYLOCOCCUS AUREUS*

H. SAITO AND H. TOMIOKA

*From the Department of Microbiology and Immunology, Shimane Medical University, Izumo* 693, *Japan*

Received 20 April 1979 Accepted 2 October 1979

**Summary.—Peritoneal macrophages from Sarcoma-180-bearing mice against *Staphylococcus aureus* were studied to determine the *in vitro* phagocytic capacities. When the phagocytic system was opsonized with normal mouse serum, macrophage phagocytic activity increased markedly soon after tumour graft and then returned to normal. A new antiphagocytic factor was detected in the serum of tumour-bearers soon after tumour implantation. This factor was of tumour origin, stable even at a temperature of 56°C for 30 min and non-dialysable. Peptone-starch-induced macrophages were less sensitive to the factor than their unstimulated counterpart.**

CONFLICTING observations have been reported on the state of macrophage phagocytic function in tumour-bearers. The carbon-clearance ability of the reticuloendothelial system, the non-specific phagocytic function performed mainly by liver macrophages, is severely repressed soon after Lewis lung carcinoma transplantation in mice (Otu *et al.*, 1977). It was reported by Gudewicz & Saba (1977) that alveolar macrophages from Walker 256 carcinosarcoma-bearing rats showed considerably depressed phagocytic activity against *Pseudomonas aeruginosa*. There is other evidence, however, that neoplastic disease induces a stimulation of macrophage phagocytic function. For instance, Meltzer & Stevenson (1978) reported that phagocytosis of IgG-coated sheep erythrocytes by macrophages was significantly augmented in tumour-bearing mice.

The present study was undertaken to determine whether or not the alteration of phagocytic capacity can be detected in macrophages from Sarcoma-180-bearing animals, using viable *Staphylococcus aureus* as the phagocytic material. We also attempted to examine whether or not changes in capacity of macrophage phagocytosis are caused by a factor of tumour origin.

We found that phagocytosis of bacteria by macrophages from tumour-bearers was remarkably enhanced in the early phase after tumour implantation. We also found a suppressive factor against macrophage phagocytic function with a mol. wt $>10,000$ in the mouse serum soon after tumour graft.

### MATERIALS AND METHODS

*Mice.*—Colony-bred DD mice of both sexes were purchased from Shizuoka Union for Experimental Animals, Shizuoka, Japan.

*Tumour.*—Sarcoma-180 (S-180) an allo-transplantable tumour kindly provided by Dr I. Umezawa (Kitasato Institute, Tokyo) was maintained by serial s.c. passage in DDY mice. The tumour was excised under aseptic conditions and finely minced in phosphate-buffered saline (PBS, pH 7·2) containing 100 μg/ml of streptomycin. The resulting cell suspension was filtered through an 80-mesh stainless-steel screen and washed once with PBS. Usually each animal was given $10^6$ tumour cells s.c. into the right flank.

*Bacterium.*—*S. aureus* Strain 209P was obtained from Dr Y. Kanemasa (Okayama University, Medical School, Okayama, Japan) and maintained in heart-infusion agar. The organism was inoculated into brain/heart infusion broth (Difco Lab., U.S.A.) and cultured at 37°C for 18–24 h. The cells were centrifuged at 2000 *g* for 15 min, washed once with PBS and finally suspended in Hanks' balanced salt solution (pH 7·4) containing 0·01% crystalline bovine serum albumin (Sigma Co., U.S.A.) and 0·0056M glucose (referred to as HBG).

*Serum.*—Serum was obtained from the normal or tumour-bearing mice by exsanguination from the heart and stored at −80°C.

*Peritoneal and peritoneal-exudate cells.*—Peritoneal-exudate cells (PEC) were obtained from mice given 5% proteose peptone–5% soluble starch i.p. 3 days before harvest by the following method. A 6ml portion of HBG containing 5 i.u./ml of heparin (Novo Co., Denmark) was injected into the abdominal cavity of each mouse. The mice were exsanguinated and 5 ml of the peritoneal fluid from each mouse was withdrawn. Samples of the peritoneal fluid from 5–15 mice were pooled, centrifuged at 200 *g* for 10 min at 4°C, treated with distilled water for 30 sec to lyse the erythrocytes, and then washed with HBG. Viability of the resultant cell preparation was estimated by the nigrosin-exclusion test to be 90–98%. Peritoneal cells (PC) were collected from untreated mice in the same way as mentioned above, and the viability was estimated at 92–98%. The cell population of PC and PEC was analysed by microscopy after Giemsa staining. PC were composed of 62–75% mononuclear phagocytes, 5–14% granulocytes, and 19–32% lymphocytes, whilst PEC consisted of 60–77% mononuclear phagocytes, 10–24% granulocytes, and 8–20% lymphocytes.

*Phagocytosis test.*—Phagocytosis was measured by two methods designated as "colony-forming unit (CFU) assay" and "microscopic assay".

CFU assay was carried out according to the method of Cohn & Morse (1959). The phagocytosing mixture (0·5 ml) in siliconized sterile tubes (10 × 100 mm) contained $1{\cdot}4\text{–}3{\cdot}8 \times 10^6$ PC or PEC, $\sim 10^5$ bacteria, sample serum, and HBG. The tubes were sealed with rubber stoppers and then incubated at 37°C on a reciprocal shaker at 160–180 cycles/min. At intervals of up to 240 min, phagocytosis was stopped by the addition of 5 ml of cold PBS containing 0·05% bovine serum albumin, followed by centrifugation at 200 *g* for 4 min. In a preliminary test, no significant reduction in the recovery of extracellular bacteria was noted after this centrifugation procedure. From the upper one third of the tube, 0·5 ml of the supernatant was withdrawn and counted for its CFU on nutrient agar plates.

For the microscopic assay, phagocytosis was performed in the same manner as described above except for the incubation mixture: $1{\cdot}2 \times 10^6$ PC or PEC, $8 \times 10^5$ bacteria, sample serum, and HBG. After phagocytosis, the cell pellet obtained by centrifugation at 200 *g* for 4 min was smeared, fixed with methanol, and stained with Giemsa solution. By light microscopy, the number of macrophage-ingested bacteria was counted, and the percentage of the phagocytosing cells to total macrophages was calculated (% phagocytosing cells). In some cases, the mean number of ingested bacteria per phagocyte was also recorded.

*S-180 tumour-cell culture.*—S-180 tumour-cell suspension prepared by the method mentioned above ("Tumour") was given i.p. at a dose of $10^6$ cells/mouse. Eight to 10 days later the ascitic cells were collected, treated with distilled water, and washed with Eagle's minimal essential medium (MEM). The resultant cells ($6 \times 10^7$) were suspended in 30 ml of MEM containing 10% foetal bovine serum (Microbiological Associates Inc., U.S.A.) in a 500ml glass culture bottle and incubated at 37°C for 1 h. Non-adherent cells were collected and the same procedure was repeated. The final cell suspension was composed mainly of tumour cells (>90%) while the remainder was mononuclear phagocytes and lymphocytes. Tumour cells were then cultured in MEM supplemented with 10% foetal bovine serum at 37°C for up to 3 days in 5% $CO_2$ and 95% humidified air.

*Calculation.*—The value of the "relative opsonization index", which means the relative opsonic activity of the sample serum as compared with normal mouse serum (NMS), was calculated as follows:

$$\text{Relative opsonization index (\%)} = \frac{\text{CFU}(-\text{serum}) - \text{CFU}(+\text{sample serum})}{\text{CFU}(-\text{serum}) - \text{CFU}(+\text{NMS})} \times 100$$

or

$$= \frac{\%\text{phag}(+\text{sample serum}) - \%\text{phag}(-\text{serum})}{\%\text{phag}(+\text{NMS}) - \%\text{phag}(-\text{serum})} \times 100$$

where CFU means extracellular CFU after phagocytosis and %phag indicates percentage of phagocytosing cells.

### RESULTS

### *Alteration in the phagocytic function of macrophages after tumour implantation*

Changes in phagocytic capacity of the peritoneal macrophages from tumour-bearers against *S. aureus* were studied after s.c. injection of S-180 tumour cells into 8-week-old male mice (8 mice per regimen). At intervals after tumour graft, PC and serum were drawn from the same mice of each experimental group, pooled, and examined for phagocytic and opsonic activities, respectively. The results are summarized in Fig. 1. When phagocytosis was performed without addition of serum, the phagocytic ability of macrophages from tumour-bearers was not altered significantly for up to 20 days after tumour implantation. In contrast, phagocytosis in the presence of NMS markedly increased on the 1st day, persisted through the 5th day, and returned to almost normal levels by the 20th day (the increases 1–5 days after tumour graft were statistically significant, $P < 0{\cdot}005$ by $\chi^2$ test). On the other hand, phagocytosis in the presence of serum from tumour bearers (S-180 MS) remarkably increased on the 1st day ($P < 0{\cdot}005$ by $\chi^2$ test), rapidly decreased by the 3rd day, remained low for 2 additional days, and then gradually returned to the 1st-day level.

These findings indicate that the phagocytic activity of macrophages against *S. aureus* is enhanced, particularly in the latent period of the tumour growth. It is also suggested that the opsonic activity of S-180 MS is reduced 3–5 days after tumour transplantation.

FIG. 1.—Changes in macrophage phagocytic function after S-180 tumour implantation in mice. (A) Peritoneal resident macrophages were obtained from tumour-bearing mice at the indicated time and examined for phagocytic capacities against *S. aureus* in the absence (△) or presence of either 10% NMS (○) or 10% S-180 MS (●) sampled at the same time. (B) Yields of total peritoneal cells (PC □) and mononuclear phagocytes (■) per g body wt, and tumour wt (▲) at the indicated time.

### *Decrease in opsonic activity of S-180 MS after tumour graft*

To ascertain the net changes in opsonizing ability of S-180 MS after tumour injection, the value of the "relative

FIG. 2.—Opsonic activity of S-180 MS as a function of time after tumour implantation. The values of "relative opsonizing index" were calculated from the data in Fig. 1, using the equation in the text. (○) 10% S-180 MS *vs* 10% NMS; (●) 10% S-180 MS + 10% NMS *vs* 20% NMS; (▲) tumour wt.

opsonization index" was calculated from the data in Fig. 1. As shown in Fig. 2, tumour implantation quickly resulted in a depressed opsonic activity of S-180 MS, the lowest level being reached on the 3rd day after tumour graft (open circles). It was also observed that the value with mixed serum (10% each of S-180 MS and NMS) changed similarly to the case of S-180 MS alone (solid circles).

*Antiphagocytic factor in S-180 MS*

The results shown in Fig. 2 suggest the presence of an antiphagocytic substance, since decreased opsonic activity of S-180 MS was not fully overcome by supplemental addition of NMS as the opsonin source. We therefore attempted to determine whether a similar phenomenon could be also observed using macrophages from normal mice by microscopic assay. As shown in Fig. 3, the phagocytic ability of peptone–starch-elicited macrophages from normal mice (open bar) was markedly suppressed when S-180 MS was added instead of NMS. This suppression was not overcome even when S-180 MS was supplemented with a sufficient amount of serum opsonins, by further addition of NMS. On the contrary, as shown also in Fig. 3, bacterial phagocytosis of polymorphonuclear leucocytes (PMN; shaded bar) was not reduced as significantly as in the case of macrophages, when S-180 MS was added instead of NMS. It is noteworthy that stimulation of PMN phagocytosis by the addition of NMS as the opsonin source was not as marked as in the case of macrophages. These observations indicate that S-180 MS contains an antiphagocytic factor or factors specifically effective on macrophages, but not on PMN.

FIG. 3.—Effect of S-180 MS on bacterial phagocytosis of peptone–starch-induced macrophages and PMN from normal mice. Phagocytosis assay was carried out by microscopy on the basis of the number of bacteria ingested per phagocyte. Each experiment was performed in duplicate and the means and variations are indicated. In this assay system, the 99% confidence limit (Student's $t$ test) was calculated as $\pm 5{\cdot}47\%$.

FIG. 4.—Effect of S-180 MS on bacterial phagocytosis of peptone–starch-induced macrophages from normal mice. Macrophage phagocytosis of *S. aureus* was performed in the absence (■) and presence of 10% NMS (○), 10% S-180 MS (△) or 10% S-180 MS plus NMS (▲). The assay was carried out by the CFU method. In the absence of macrophages (□), bacterial CFU did not change significantly during the incubation time.

The bar indicates the 95% confidence limit (Student's $t$ test).

As illustrated in Fig. 4, the phagocytic ability of PEC from normal mice given peptone–starch 3 days before harvest (measured by CFU assay) was also markedly reduced when S-180 MS was added instead of NMS (open triangles *vs* open circles). In this case, PEC were composed of 70% macrophages, 14% PMN, and 16% lymphocytes. Therefore, using the data presented in Fig. 3, it may be estimated that about 8·3% of the total bacterial phagocytosis of the PEC in the presence of 10% NMS is due to contaminating PMN. Similarly, when 10% S-180 MS was added instead of NMS, the contaminating PMN can be regarded as the cause of about 16% of the total phagocytosis. However, since the present antiphagocytic factor specifically acts on macrophages but not on PMN as indicated in Fig. 3, the reduction of bacterial phagocytosis observed here can be regarded as mainly due to the depression of the phagocytic capacity of macrophages, but not of PMN. Furthermore, there was no reversal of the reduction of macrophage phagocytosis when S-180 MS was supplemented with a further addition of NMS as the opsonin source (solid triangles). This indirectly suggests that the reduced macrophage phagocytic capacity in the presence of S-180 MS is not corrected by supplemental addition of NMS, because the bacterial phagocytic activity of PMN did not differ significantly between the cases where S-180 MS was added alone or with supplemental NMS, as shown in Fig. 3. These observations indicate again that S-180 MS has a suppressive factor against macrophage phagocytic function.

It was also noted that S-180 MS exhibited a dose-dependent suppression of the bacterial phagocytosis of macrophages (data not shown).

*Stimulated macrophages have a reduced sensitivity to the antiphagocytic factor*

As can be seen in the first column of Table I (Experiment 1) percentage reductions in "relative opsonization index" (which means the sensitivity of macrophages to the action of the present antiphagocytic factor) were 63 ± 20 and 29 ± 4% for resident and peptone–starch-stimulated macrophages, respectively. In this case, bacterial phagocytosis tests were performed on PC and PEC from normal mice by the CFU assay. The PC and PEC used here were found to be composed of 70 and 65% mononuclear phagocytes, and to be contaminated with 7·3 and 10% PMN respectively. Therefore it seems that bacterial phagocytosis due to contaminating PMN dose not exceed 10% of total phagocytosis in both cases, at most, since PMN phagocytic capacity was found to be less than (or equal to) that of macrophages in the presence of various serum

TABLE I.—*Comparison of resident and peptone–starch-induced macrophages as related to sensitivity to the antiphagocytic factor of S-180 MS*

| Exp. | Macrophage | Macrophage donor | Serum addition | % Reduction of opsonic activity[a] | Relative sensitivity[b] | Assay |
|---|---|---|---|---|---|---|
| 1 | Resident | Normal mice | 10% S-180MS* | 63 ± 20 | | CFU |
| | Peptone† | Normal mice | 10% S-180MS | 29 ± 4 | 46 | |
| 2 | Resident | S-180 mice‡ | 10% S-180MS | 98 ± 3 | | Microscopy |
| | Peptone | S-180 mice | 10% S-180MS | 71 ± 2 | 72 | |
| 3 | Resident | S-180 mice | 10% S-180MS + 10% NMS | 58 ± 13 | | Microscopy |
| | Peptone | S-180 mice | 10% S-180MS + 10% NMS | 34 ± 17 | 59 | |

[a] % Reduction of opsonic activity = 100 − "relative opsonization index (%)" of sample serum. The mean and variation of two observations are presented.

[b] Relative sensitivity of each kind of macrophage to the antiphagocytic factor, taking the values of resident macrophages as 100.

* S-180 MS was obtained 3 days after tumour implantation.

† Peptone–starch-induced macrophage.

‡ S-180 tumour-bearing mice, 3 days after tumour graft.

supplements as shown in Fig. 3. Moreover, as can be seen in Fig. 3, the present antiphagocytic factor in S-180 MS exhibits none of the significant suppressive actions against PMN phagocytic function. Thus the reductions in opsonic activity observed here for PC and PEC can be regarded as representing mainly depression of macrophage phagocytic ability by the antiphagocytic factor, but not of PMN phagocytosis. Similar results were obtained in separate experiments by microscopic assay using PC and PEC from S-180 tumour-bearing mice (Exps 2 and 3). These findings suggest that macrophages stimulated with peptone–starch are more resistant to the inhibitory action of the antiphagocytic factor than their unstimulated counterparts. It may be worth noting that sensitivity of macrophages from S-180-tumour-bearing animals to the antiphagocytic factor is considerably greater than that of macrophages from normal donors (Exps 1 & 2). However, in a separate experiment, it was found by microscopic assay that the % reduction of opsonic activity of peptone–starch-induced macrophages from normal mice was $55 \pm 12\%$. This value is not much lower than the value, $71 \pm 2\%$, obtained for peptone–starch-elicited macrophages from S-180 tumour-bearing mice. Thus it seems most likely that the difference between both types of macrophages from S-180 tumour bearers and normal mice in their sensitivities to the antiphagocytic factor simply reflects the different assay systems used, rather than a real effect.

The results are given in Table II. When 10% of S-180 MS was added to the phagocytozing incubation mixture (using peptone–starch-induced PEC from normal mice as phagocytes) with a supplementary addition of an equal amount of NMS, the extracellular CFU after 2h phagocytosis was nearly twice that of the control (20% NMS added) indicating the antiphagocytic action of S-180 MS. A similar degree of antiphagocytic activity was also noted when 10% S-180 MS which had been thoroughly dialysed against PBS, in place of untreated S-180 MS, was added to the phagocytosis system. Thus, it appears that the antiphagocytic factor does not pass through the dialysis membrane. It was also found that addition of 10% of heated S-180 MS (56°C for 30 min) produced a remarkable reduction in phagocytosis, whereas only a slight decrease was obtained when 10% of NMS similarly treated was added. This indicates that the antiphagocytic factor is stable to heat treatment at 56°C for 30 min. This factor could be precipitated at 50% saturation of ammonium sulphate.

TABLE II.—*Effect of physical treatments on antiphagocytic activity of S-180 MS*

| Serum addition | Extracellular CFU per tube ($\times 10^3$)[a] |
|---|---|
| 20% NMS | $1{\cdot}70 \pm 0{\cdot}23$ |
| 10% NMS + 10% S-180 MS | $2{\cdot}98 \pm 0{\cdot}33$ |
| 10% NMS + 10% dialysed S-180 MS* | $2{\cdot}88 \pm 0{\cdot}26$ |
| 10% NMS + 10% heated NMS† | $1{\cdot}73 \pm 0{\cdot}69$ |
| 10% NMS + 10% heated S-180 MS† | $3{\cdot}40 \pm 0{\cdot}40$ |

[a] Number of viable extracellular staphylococci per tube after 2h phagocytosing incubation. The mean values of 4 observations of experimental data ± s.e. (Student's *t* test) are indicated.

* S-180 MS obtained 3 days after tumour graft was dialysed against 100 vol of PBS overnight.

† 56°C for 30 min.

*Origin of the antiphagocytic factor*

In attempts to determine the origin of this antiphagocytic factor, we investigated whether or not a similar factor is produced by S-180 tumour cells *in vitro*. Three-day cultured fluid of S-180 tumour cells was dialysed against PBS and fractionated with 50%-saturated ammonium sulphate. The precipitate was redissolved in PBS, dialysed against the same buffer, and then made up to 1/15 volume of starting culture fluid. This was sterilized for 5 min by UV-irradiation using a 15W UV lamp at 20 cm.

As indicated in Fig. 5, phagocytic ability of peptone–starch-induced macrophages from normal mice linearly decreased as the amount of fraction obtained from S-180 cell culture fluid increased.

FIG. 5.—Dose response of antiphagocytic activity of the fraction obtained by ammonium sulphate precipitation at 50% saturation from 3-day-old S-180 tumour-cell culture fluid. Each plot and its bar indicate the mean of 4 observations and the 95% confidence limit (Student's *t* test), respectively.

Thus, the antiphagocytic factor is apparently produced in the culture fluid of S-180 tumour cells. It should be noted that the fraction exhibited antiphagocytic activity even after UV irradiation, indicating that the antiphagocytic action of S-180 cell culture fluid is not virally mediated. Moreover, this is supported by the observation that both S-180 MS and culture fluid of S-180 cells heated at 100°C for 5 min showed the same level of antiphagocytic activity as that of untreated preparations. Spleen cells from normal mice were also found to produce an antiphagocytic substance, but the amount was considerably lower than that from S-180 cells (data not shown).

### DISCUSSION

The objective of the present work was to determine whether or not alteration of the phagocytic capacity of macrophages from S-180 tumour-bearing mice can be detected in the *in vitro* bacterial phagocytosis system. The following conclusions were drawn.

The s.c. implantation of the tumour in mice induced a marked increase in macrophage phagocytosis of *S. aureus* in the presence of NMS, as an opsonin source. This was noted particularly in the latent period of tumour growth. A similar result was obtained when the mean number of bacteria ingested per macrophage was estimated instead of "% of phagocytosing cells" (data not shown). (In these cases phagocytosis was performed under suboptimal conditions.) Accordingly, it seems that this early increase in bacterial phagocytosis of macrophages from tumour-bearing mice is due to both the increase in the cell population of highly phagocytic macrophages and a certain enhancement in phagocytic capacity of individual phagocytes. A similar observation has been described concerning macrophage phagocytosis of IgG-coated sheep erythrocytes in cases of Fibrosarcoma 1038 and Hepatoma 129 (Meltzer & Stevenson, 1978).

The serum from tumour-bearing mice showed a remarkable reduction in opsonizing activity soon after tumour graft. This situation can be mainly attributed to the presence of some antiphagocytic factor of tumour origin in S-180 MS, since the supplementary addition of NMS (as an opsonin source) into the phagocytic system failed to reverse the depression of opsonic activity in S-180 MS. However, it is not clear whether this antiphagocytic factor merely acts as a competitive inhibitor against serum opsonins or actually alters the macrophage phagocytic function.

The present antiphagocytic factor was stable to heat treatment at 56°C for 30 min and non-dialysable through dialysis membrane. Thus, it probably has a mol. wt > 10,000. In addition, it was observed that cultured S-180 tumour cells actually produced a similar antiphagocytic factor. Accordingly, the antiphagocytic factor detected in S-180 MS is considered to originate from S-180 tumour cells *per se*.

The present antiphagocytic factor of S-180 tumour origin seems to differ from the humoral factor of Hepatoma 129 origin which has an inhibitory activity on macrophage chemotaxis (Pike & Snyderman, 1976) or the factor of SA1 spindle-cell sarcoma origin with a suppressive activity against macrophage bactericidal function (North *et al.*, 1976*a*). Both these factors are dialysable and their mol. wts are considered to be < 10,000. It is noteworthy that the antiphagocytic factor of S-180 tumour origin showed no significant inhibitory action against macrophage staphylocidal activity (data not shown).

The depressed state of opsonizing ability of S-180 MS 3–5 days after tumour graft was subsequently restored, and replaced by a contrasting state, somewhat more active than NMS. As indicated by North *et al.* (1976*b*, 1977) it seems likely that this phenomenon coincided with the expression of T-cell-mediated concomitant immunity to tumour in the recipient mice, *i.e.* the appearance of T-cell-derived stimulating factor(s) of macrophage phagocytosis in the serum of S-180 tumour-bearers after the latent period of tumour growth.

As indicated in Table I, peptone–starch-induced macrophages were more resistant to the antiphagocytic action of the present S-180 tumour factor than the resident macrophages. This suggests that stimulation of macrophages with certain agents can overcome the subversion of the phagocytic function by the tumour factor.

Our results may assist in elucidating the conflicting data of Meltzer & Stevenson (1978) and Otu *et al.* (1977) concerning the macrophage phagocytic function in tumour-bearers. The former found in *in vitro* studies that macrophage phagocytosis of IgG-coated erythrocytes was markedly increased in cases of Fibrosarcoma 1038- and Hepatoma 129-bearing mice. It was also reported that the tumour serum failed to suppress the macrophage phagocytic function when the serum was given to normal recipients. On the contrary, Otu *et al.* (1977) reported that host carbon clearance ability, a nonspecific phagocytic activity of mainly liver macrophages, was considerably reduced early after Lewis lung carcinoma implantation in mice. They also found that the serum from tumour-bearers caused a significant decrease in carbon clearance ability of the recipient mice. In S-180 tumour-bearing mice, the phagocytic capacity of macrophages from tumour-bearers was enhanced significantly in the latent period of tumour growth, when the *in vitro* phagocytosis was performed in the presence of a sufficient amount of NMS as the opsonin source. In addition, a large amount of antiphagocytic factor was produced in the serum of tumour-bearing mice also in the latent period of tumour growth. These observations strongly suggest that the stimulation of macrophage phagocytic function soon after tumour graft actually occurs in all the cases, whereas the amount of antiphagocytic factor of tumour origin differs considerably between types of tumours. In the cases of Fibrosarcoma 1038 and Hepatoma 129 (Meltzer & Stevenson, 1978) the failure of the tumour sera to depress the macrophage phagocytic ability of the recipient animals indicates a small amount of antiphagocytic factor. In contrast, it appears that a large amount of antiphagocytic factor is produced in the serum of Lewis lung carcinoma-bearing mice as this tumour serum given to normal mice caused a marked reduction in the carbon-clearance ability of liver macrophages of the recipients (Otu *et al.*, 1977). Therefore the apparent reduction in the carbon-clearance ability of Lewis lung carcinoma-bearers seems to be due to the antiphagocytic effect of the serum factor of tumour origin rather than to a decrease in the macrophage phagocytic function *per se*.

In any case, the apparent alteration in macrophage phagocytic capacity after tumour transplantation probably depends on the amount (activity) of antiphagocytic factor in the serum of tumour-bearing animals. However, there remains the possibility that the contradictory situations mentioned above are merely due to the different phagocytic particles used in the above studies.

Purification of the antiphagocytic factor of S-180 tumour origin is now under way, and results will be reported elsewhere.

### REFERENCES

COHN, Z. A. & MORSE, S. I. (1959) Interaction between rabbit polymorphonuclear leucocytes and staphylococci. *J. Exp. Med.*, **110**, 419.

GUDEWICZ, P. W. & SABA, T. M. (1977) Inhibition of phagocytosis and glucose metabolism of alveolar macrophages during pulmonary tumour growth. *Br. J. Cancer*, **36**, 670.

MELTZER, M. S. & STEVENSON, M. M. (1978) Macrophage function in tumour-bearing mice: dissociation of phagocytic and chemotactic responsiveness. *Cell. Immunol.* **35**, 99.

NORTH, R. J., KIRSTEIN, D. P. & TUTTLE, R. L. (1976*a*) Subversion of host defense mechanisms by murine tumours. I. A circulating factor that suppresses macrophage-mediated resistance to infection. *J. Exp. Med.*, **143**, 559.

NORTH, R. J., KIRSTEIN, D. P. & TUTTLE, R. L. (1976*b*) Subversion of host defense mechanisms by murine tumors. II. Counter-influence of concomitant antitumor immunity. *J. Exp. Med.*, **143**, 574.

NORTH, R. J. & KIRSTEIN, D. P. (1977) T-cell mediated concomitant immunity to syngeneic tumors. I. Activated macrophages as the expressors of nonspecific immunity to unrelated tumors and bacterial parasites. *J. Exp. Med.*, **145**, 275.

OTU, A. A., RUSSELL, R. J., WILKINSON, P. C. & WHITE, R. G. (1977) Alterations of mononuclear phagocyte function induced by Lewis lung carcinoma in C57BL mice. *Br. J. Cancer*, **36**, 330.

PIKE, M. C. & SNYDERMAN, R. (1976) Depression of macrophage function by a factor produced by neoplasms: a mechanism for abrogation of immune surveillance. *J. Immunol.*, **117**, 1243.